# Supplementary material for: “We’re very much part of the team here”: A culture of respect for Indigenous health workforce transforms Indigenous health care
Source: PLoS One. 2020 Sep 22;15(9):e0239207. doi: 10.1371/journal.pone.0239207 (PMC7508383; doi:10.1371/journal.pone.0239207)
Supplement: Attachment — Submitted filename: review workforce.pdf [file pone.0239207.s001.pdf]

Review report

Abstract: the abstract is sound.

Introduction: it would be good to put more context on the profile of Aboriginal peoples then connect this to cancer. Cancer is higher in our communities, however there needs info on the factors, including risk factors, that lead to this.

Methodology: as an Aboriginal scholar, I would like to know this research embodied the principals and practices of Indigenous Research methodology and Indigenous standpoint theory. The theory lacks any theoretical knowledge on methodology. How did Aboriginal people have governance of the study? How were member checks done? How were they involved in the analysis and interpretation of the data? Was the research team filled with non-indigenous researchers? If so, how did they exercise cultural safety? Given they interviewed cancer patients and staff, how did they manage GL&T associated with cancer and dying? Currently, this paper only tells the methods, without the science behind it. Also, provide a profile of the sites that does not disclose who they are. Why were the interviews done over a long timeframe? Could the reasons be averted?

Findings: Currently, the paper needs a deeper analysis and reporting of the data findings. Currently, as it stands, it reads like a carefully crafted government report: good things at the start and one section on things to build upon. This paper needs the data in the final section, gaps and continuous improvement, to be located under the other headings. For example, paragraph two could go under the section on employment.

The section read overly positive. The reading of the literature does not stack up to this research paper.

Discussion: the discussion just repeats the findings with limited interrogation of the data relating to the literature. It is hard to believe that these two agencies do not have challenges.
